# Efavirenz and Metabolites in Cerebrospinal Fluid: Relationship with *CYP2B6* c.516G→T Genotype and Perturbed Blood-Brain Barrier Due to Tuberculous Meningitis

**DOI:** 10.1128/AAC.00280-16

**Published:** 2016-07-22

**Authors:** Sam Nightingale, Tran Thi Hong Chau, Martin Fisher, Mark Nelson, Alan Winston, Laura Else, Daniel F. Carr, Steven Taylor, Andrew Ustianowski, David Back, Munir Pirmohamed, Tom Solomon, Jeremy Farrar, M. Estée Törok, Saye Khoo

**Affiliations:** a Institute of Infection and Global Health, University of Liverpool, Liverpool, United Kingdom; b Department of Molecular and Clinical Pharmacology, University of Liverpool, Liverpool, United Kingdom; c Royal Liverpool and Broadgreen University Hospitals NHS Trust, Liverpool, United Kingdom; d Oxford University Clinical Research Unit, Hospital for Tropical Diseases, Ho Chi Minh City, Vietnam; e Brighton and Sussex University Hospitals NHS Trust, Brighton, United Kingdom; f St. Stephen's AIDS Research Trust and Chelsea and Westminster Hospital NHS Foundation Trust, London, United Kingdom; g St. Mary's Hospital, Imperial College Healthcare NHS Trust, London, United Kingdom; h Birmingham Heartlands Hospital, Heart of England NHS Foundation Trust, Birmingham, United Kingdom; i North Manchester General Hospital, Pennine Acute Hospitals NHS Trust, Manchester, United Kingdom; j Walton Centre for Neurology and Neurosurgery, Liverpool, United Kingdom; k Centre for Tropical Medicine, University of Oxford, Oxford, United Kingdom; l University of Cambridge, Department of Medicine, Cambridge, United Kingdom; m Cambridge University Hospitals, NHS Foundation Trust, Cambridge, United Kingdom; n Public Health England, Clinical Microbiology and Public Health Laboratory, Cambridge, United Kingdom

## Abstract

Efavirenz (EFZ) has been associated with neuropsychiatric side effects. Recently, the 8-hydroxy-EFZ (8OH-EFZ) metabolite has been shown to be a potent neurotoxin *in vitro*, inducing neuronal damage at concentrations of 3.3 ng/ml. EFZ induced similar neuronal damage at concentrations of 31.6 ng/ml. We investigated the effect of genotype and blood-brain barrier integrity on EFZ metabolite concentrations in cerebrospinal fluid (CSF). We measured CSF drug concentrations in subjects from two separate study populations: 47 subjects with tuberculous meningitis (TBM) coinfection in Vietnam receiving 800 mg EFZ with standard antituberculous treatment and 25 subjects from the PARTITION study in the United Kingdom without central nervous system infection receiving 600 mg EFZ. EFZ and metabolite concentrations in CSF and plasma were measured and compared with estimates of effectiveness and neurotoxicity from available published *in vitro* and *in vivo* data. The effect of the *CYP2B6* c.516G→T genotype (GG genotype, fast EFV metabolizer status; GT genotype, intermediate EFV metabolizer status; TT genotype, slow EFV metabolizer status) was examined. The mean CSF concentrations of EFZ and 8OH-EFZ in the TBM group were 60.3 and 39.3 ng/ml, respectively, and those in the no-TBM group were 15.0 and 5.9 ng/ml, respectively. Plasma EFZ and 8OH-EFZ concentrations were similar between the two groups. CSF EFZ concentrations were above the *in vitro* toxic concentration in 76% of samples (GG genotype, 61%; GT genotype, 90%; TT genotype, 100%) in the TBM group and 13% of samples (GG genotype, 0%; GT genotype, 18%; TT genotype, 50%) in the no-TBM group. CSF 8OH-EFZ concentrations were above the *in vitro* toxic concentration in 98% of the TBM group and 87% of the no-TBM group; levels were independent of genotype but correlated with the CSF/plasma albumin ratio. Potentially neurotoxic concentrations of 8OH-EFZ are frequently observed in CSF independently of the *CYP2B6* genotype, particularly in those with impaired blood-brain barrier integrity.

## INTRODUCTION

Despite concerns over central nervous system (CNS) toxicity, efavirenz (EFZ) is widely deployed within first-line combination HIV treatment regimens worldwide because of its effectiveness, established safety record, and resilience to hepatic enzyme induction by rifampin in patients who require concomitant therapy against tuberculosis (TB) (1, 2). EFZ undergoes rapid absorption, with maximum plasma concentrations being reached in 3 to 6 h and therapeutic levels being achieved within a few days of the commencement of treatment (3). There is large interindividual variability in EFZ pharmacokinetics (4–7), placing patients with low plasma concentrations at risk of losing virological control and developing resistance and those with high plasma concentrations at risk of developing adverse effects (8, 9). EFZ is primarily metabolized by cytochrome P450 *CYP2B6* to yield the most abundant metabolite, 8-hydroxy-EFZ (8OH-EFZ). Comparatively minor alternative metabolic pathways are through *CYP2A6* (leading to the 7OH-EFZ metabolite) and *CYP3A* (10).

EFZ plasma concentrations relate strongly to the genetic polymorphism in *CYP2B6* metabolism (11–15), including the most commonly studied *CYP2B6* single nucleotide polymorphism c.516G→T (rs3745274), which encodes a Gln172His amino acid substitution. The *CYP2B6* c.516G→T GG genotype is associated with a fast EFV metabolizer status, the GT genotype is associated with an intermediate metabolizer status, and the TT genotype is associated with a slow metabolizer status. Preliminary data suggest that in *CYP2B6* slow metabolizers, *CYP2A6* represents the dominant route of elimination and may be affected by enzyme inhibition through concomitant isoniazid administration (16). This may have pharmacogenetic implications, as *CYP2A6* has considerable copy number variation in Southeast Asian populations (17). The effect of the *CYP2A6* copy number on CSF EFZ and metabolite concentrations in those with and without slow *CYP2B6* metabolizer status is not known.

The results of *in vitro* experiments indicate that 8OH-EFZ is associated with cytotoxicity via stimulation of mitochondrial dysfunction and stress-activated signaling pathways (18). In addition, 8OH-EFZ has been shown to be prone to oxidative degradation with potentially toxic quinone-imine derivatives (19). Recently, 8OH-EFZ was shown to be neurotoxic *in vitro* at a concentration similar to the concentrations found in cerebrospinal fluid (CSF) (20). That study demonstrated that 8OH-EFZ concentrations of just 3.3 ng/ml caused neuronal damage, inducing calcium flux, apoptosis, and considerable damage to dendritic spines. These changes were not observed for EFZ or 7OH-EFZ at this level. Concentrations of EFZ and 7OH-EFZ approximately 10 times the concentration of 8OH-EFZ were required to induce similar damage. The role of 8OH-EFZ in EFZ-associated CNS toxicity has not been elucidated.

In this study, we developed sensitive, accurate, and precise assays for measuring EFZ and its metabolites in CSF. We aimed to characterize the disposition of EFZ and its metabolites within CSF in HIV-infected patients with and without tuberculous meningitis (TBM) and to evaluate the impact of pharmacogenetic variability on drug disposition.

## MATERIALS AND METHODS

### Participants and sampling.

The CSF pharmacokinetics of EFV were studied in two separate patient populations. Since these cohorts differ in several characteristics, no statistical comparisons between the two groups were undertaken.

### (i) TBM group.

In Vietnam, HIV-infected patients over 15 years of age with newly diagnosed TBM (Clinical Trials Registration number ISRCTN63659091) were randomized to receive immediate (within 7 days) or deferred (after 2 months) initiation of antiretroviral therapy as previously described (21, 22). Among the subjects in this cohort, paired CSF and blood samples were available at steady state for 47 subjects while they were receiving EFZ (>10 days) (23). Sampling was a mean of 97 days after the commencement of treatment. EFZ was dosed at 800 mg together with zidovudine plus lamivudine in a fixed-dose combination. Antituberculous therapy comprised isoniazid (5 mg/kg of body weight/day; maximum, 300 mg), rifampin (10 mg/kg/day; maximum, 600 mg), pyrazinamide (25 mg/kg/day; maximum, 2 g), and ethambutol (20 mg/kg/day; maximum, 1.2 g) for 3 months, followed by isoniazid plus rifampin for 6 months. Unless contraindicated, all patients received dexamethasone as described elsewhere (24). The mean age was 30 years (standard deviation [SD], 5.4 years), and the median CD4 cell count at the time of sampling was 81 cells/mm^3^ (interquartile range [IQR], 46 to 159 cells/mm^3^). All patients were of Southeast Asian ethnicity. Ethics approval was obtained from the Oxford Tropical Research Ethics Committee and the Hospital for Tropical Diseases Scientific and Ethical Committee.

### (ii) No-TBM group.

In the United Kingdom, paired plasma and CSF samples were obtained at a single time point from 25 subjects without CNS infection from the United Kingdom PARTITION (Penetration of Antiretroviral Therapy into the Nervous System) study (25). Participants were HIV-1-infected adults (over 16 years of age) prospectively enrolled from 2 groups: those undergoing lumbar puncture for a clinical indication or those with a history of unexplained intermittently or persistently detectable plasma HIV-1 RNA within the past 12 months. In all patients, the treating clinician believed that the CNS infection had been excluded on the basis of CSF testing and clinical findings. All patients received 600 mg of EFZ once daily; in 25 subjects, this was with tenofovir and emtricitabine, in 1 subject this was with lamivudine and abacavir, and in 1 subject this was with darunavir and ritonavir. The mean age was 46 years (SD, 8.6 years), and the median CD4 cell count at the time of sampling was 432 cells/mm^3^ (IQR, 292 to 649 cells/mm^3^). Twenty (80%) subjects were of white ethnicity, 3 (12%) were of black ethnicity, and 2 (8%) were of Asian ethnicity. No subject was receiving antituberculous therapy or other enzyme-inducing medication at the time of sampling. The study was approved by the North Wales Research Ethics Committee (Central and East).

### EFZ and metabolite measurement.

EFZ concentrations in plasma and CSF samples from subjects receiving EFZ were determined at steady state (>10 days) (23), and samples were collected at a middosing interval. EFZ metabolite concentrations in a single paired CSF-plasma sample were determined for each subject. Measurements were repeated with and without β-glucuronidase in the TBM group to determine the amount of glucuronidated compound versus the amount of free compound. The ratio between the albumin concentration in CSF and that in plasma or serum was determined as a marker of blood-brain barrier integrity.

EFZ concentrations in plasma and CSF were measured by a validated tandem liquid chromatography (LC)-mass spectrometry (MS) method as previously described (26). Freshly prepared standards, quality control samples (prepared in artificial CSF), and clinical samples (100 μl) were transferred into 7-ml stoppered glass tubes to which 100 μl of acetonitrile was added. The samples were evaporated to dryness at room temperature in a stream of nitrogen. The samples were then incubated at 37°C for 2 h with 400 μl of a solution containing 200 units of β-glucuronidase from Helix pomatia in 0.2 M sodium acetate buffer (pH 5) (27). The samples were subsequently alkalinized with 20 μl of potassium carbonate buffer (0.1 M, pH 9.4) and extracted with 3 ml of a mixture of organic solvents, ethyl acetate-hexane (60:40, vol/vol). After centrifugation, the organic phase was evaporated to dryness, the residue was reconstituted in 100 μl of a mobile phase (50/50 [vol/vol] acetonitrile-H_2_O in 1 mM ammonium acetate), and 20 μl of this solution was analyzed directly by LC-MS/MS on a Thermo Access triple-quadrupole mass spectrometer. Hexobarbital was used as the internal standard. Gradient elution was on a reverse-phase C_18_ column using 1 mM ammonium acetate in water and acetonitrile. Quantification was by selective reaction monitoring in the negative ionization mode. Accuracy and precision were satisfactory, with a mean bias of 4.8% and an intra-assay coefficient of variability of 6.5%.

### Albumin ratio.

Albumin concentrations in CSF and blood (plasma/serum) were determined by radial immunodiffusion (Bindarid). A CSF/blood albumin ratio indicative of a breach in integrity of the blood-brain barrier was taken to be ≥6.8 for subjects less than 45 years old and ≥10.2 for subjects over 45 years old (28).

### Neurotoxic concentrations.

The measured plasma and CSF concentrations were compared to the following concentrations associated with neurotoxicity. Plasma EFZ concentrations of greater than 4,000 ng/ml are associated with an increased risk of CNS side effects (8). Plasma EFZ concentrations of less than 1,000 ng/ml have historically been associated with virological failure (8). The concentrations of EFZ, 8OH-EFZ, and 7OH-EFZ associated with neuronal damage *in vitro* were 31.6, 3.3, and 33.2 ng/ml, respectively (20).

### Genetic analysis.

Genomic DNA was purified from whole blood using standard phenol-chloroform extraction methods. Allelic discrimination was performed by TaqMan real-time PCR for determination of the *CYP2B6* c.516G→T genotype and *CYP2A6* copy number using validated commercially available assays (Life Technologies, Paisley, United Kingdom).

### Statistical analysis.

The geometric mean log_10_ drug/metabolite concentrations were compared using Student's *t* test and one-way analysis of variance (ANOVA). The Pearson *r* coefficient was used to determine the correlation between continuous variables. The CD4 count and CSF/plasma ratio of the EFZ concentration were nonparametrically distributed and analyzed using the Mann-Whitney U test. Fisher's exact and chi-square tests were used for categorical demographic data. All analyses were performed using SPSS (version 22) software.

## RESULTS

**FIG 1:**
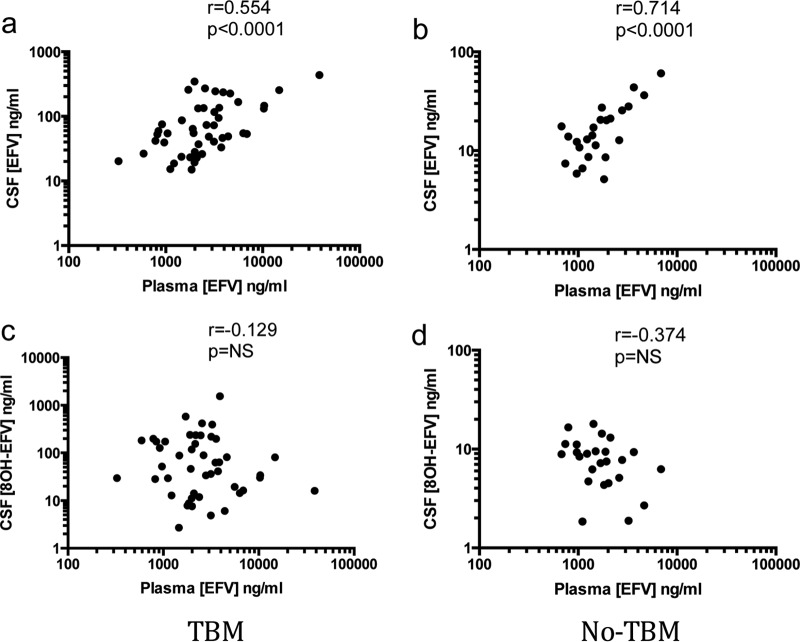
Relationship between concentrations of EFZ in plasma (a readily accessible and more easily measured parameter) and concentrations of EFZ and 8OH-EFZ in CSF. CSF and plasma EFZ concentrations were correlated in the TBM group (a) and the no-TBM group (b). No relationship was seen for 8OH-EFZ in either the TBM group (c) or the no-TBM group (d). NS, not significant.

Plasma EFZ concentrations correlated with CSF EFZ concentrations in both groups; however, there was no correlation between plasma EFZ and CSF 8OH-EFZ concentrations (Fig. 1). The median ratio of the CSF/plasma EFZ concentration was 0.027 (IQR, 0.013 to 0.056) in the TBM group and 0.010 (IQR, 0.007 to 0.012) in the no-TBM group.

### *CYP2B6* genotype.

**FIG 2:**
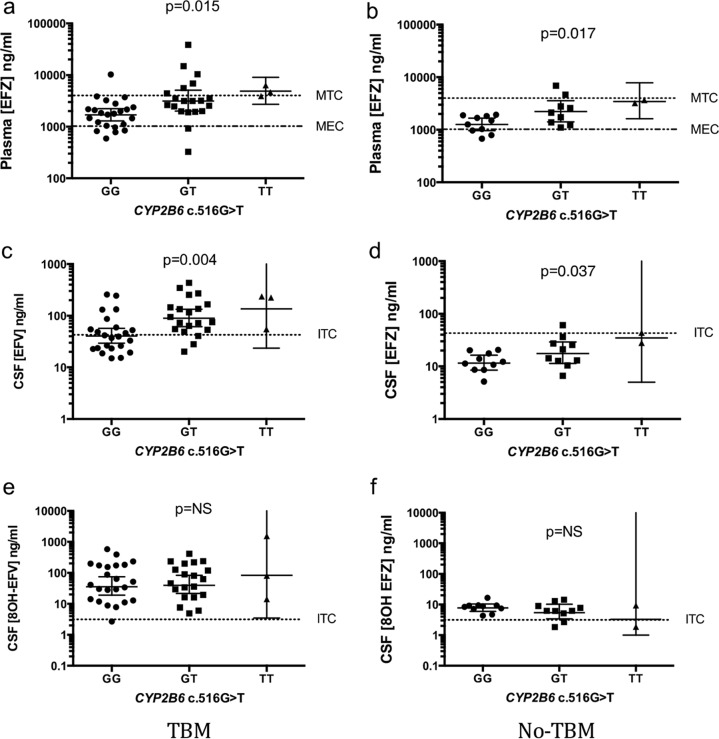
Effect of *CYP2B6* genotype on estimated effective and toxic concentrations of EFZ in plasma (a and b), EFZ in CSF (c and d), and total 8OH-EFZ in CSF (e and f). Error bars are geometric means and 95% confidence intervals for the GG/GT genotype and the geometric mean and range for the TT genotype. MTC, minimum toxic concentration; MEC, minimum effective concentration; ITC, *in vitro* toxic concentration.

Forty-six samples from the TBM group and 23 samples from the no-TBM group were successfully genotyped for *CYP2B6* c.516G→T (call rates, 98% and 88%, respectively). Allele frequencies were 50% GG genotype, 43% GT genotype, and 7% TT genotype in the TBM group and 43% GG genotype, 48% GT genotype, and 9% TT genotype in the no-TBM group (Table 1). Only 5 patients had the TT (i.e., slow metabolizer) genotype. *CYP2B6* c.516G→T was in Hardy-Weinburg equilibrium in both groups (*P* = 0.912 for the TBM group and 0.672 for the no-TBM group). The *CYP2B6* c.516G→T genotype related to the concentration of EFZ in CSF and plasma in both groups. This relationship was not present for the concentrations of the 8OH-EFZ metabolite (Table 1). The concentrations of 7OH-EFZ in plasma and CSF were also not related to genotype. There was no difference in the CSF/plasma EFZ concentration ratio according to genotype. The effect of the *CYP2B6* genotype on EFZ and 8OH-EFZ concentrations with respect to the estimated therapeutic range in plasma and the *in vitro* toxic concentrations in CSF are shown in Fig. 2. The number and proportion of CSF samples with concentrations above estimated *in vitro* toxic concentrations are given in Table 2.

**TABLE 1:** *CYP2B6* c.516G→T allele frequency and EFZ and 8OH-EFZ concentrations in CSF and plasma

*CYP2B6* c.516G→T genotype	Allele frequency (no. [%] of subjects)		Geometric mean concn^*a*^ (95% CI)							
			Plasma				CSF			
			EFZ		8OH-EFZ		EFZ		8OH-EFZ	
	TBM	No TBM	TBM	No TBM	TBM	No TBM	TBM	No TBM	TBM	No TBM
All	46 (100)	23 (100)	2,355,0 (1,836.5–3,047.9)	1,766.0 (1,383.6–2,280.3)	1,199.5 (706.3–2,128.1)	1,194.0 (883.1–1,636.8)	60.3 (46.6–79.4)	15.0 (11.7–19.7)	39.3 (25.7–63.4)	5.9 (4.4–8.2)
GG	23 (50.0)	10 (43.5)	1,694.3 (1,297.2–2,233.6)	1,264.7 (963.8–1,674.9)	1,901.1 (1,396.4–2,630.3)	1,559.6 (1,002.3–2,494.6)	40.4 (29.4–57.0)	11.5 (8.5–16.3)	35.5 (19.1–74.8)	7.8 (6.0–10.5)
GT	20 (43.5)	11 (47.8)	3,140.5 (1,995.3–5,081.6)	2,202.9 (1,482.5–3,342.0)	779.8 (269.8–2,766.9)	1,032.8 (632.4–1,749.8)	89.3 (61.5–134.0)	17.0 (11.5–26.6)	39.8 (21.6–82.8)	5.3 (3.5–9.2)
TT	3 (6.5)	2 (8.7)	4,852.9 (2,716.4–9,036.5)	3,435.6 (1,625.5–7,834.3)	666.8 (18.9–1.8 × 10^6^)	687.1 (15.8–5.2 × 10^6^)	136.1 (23.6–2,084.5)	34.8 (5.0–2,546.8)	82.8 (3.5–5.7 × 10^6^)	3.3 (1.0–>10 × 10^6^)
*P* value^*b*^			0.015	0.013	NS	NS	0.004	0.037	NS	NS

a All concentrations are with β-glucuronidase. EFZ, efavirenz.

b *P* values were determined by ANOVA. NS, not significant.

**TABLE 2:** Proportion of CSF samples with EFZ and 8OH-EFZ concentrations above *in vitro* toxic concentrations^*a*^

Drug and group	No. (%) of CSF samples from patients with the following *CYP2B6* c.516G→T genotype:			
	All	GG	GT	TT
EFZ				
TBM	35 (76)	14 (61)	18 (90)	3 (100)
No TBM	3 (13)	0 (0)	2 (18)	1 (50)
8OH-EFZ				
TBM	45 (98)	22 (96)	20 (100)	3 (100)
No TBM	20 (87)	10 (100)	9 (82)	1 (50)

a *In vitro* toxic concentrations are 31.6 ng/ml for EFZ and 3.3 ng/ml for 8OH-EFZ.

Plasma EFZ concentrations were similar between the TBM and no-TBM groups and mostly fell within the estimated therapeutic range, regardless of genotype. CSF EFZ concentrations exceeding the estimated *in vitro* neurotoxic level were observed mainly in the TBM group, particularly in those with one or more *CYP2B6* c.516G→T mutations (i.e., those with the GT or TT genotype, corresponding to intermediate or slow EFZ metabolizers, respectively). CSF 8OH-EFZ concentrations tended to be above the estimated *in vitro* neurotoxic level in both groups regardless of genotype.

### *CYP2A6* copy number variation.

Forty-six samples in the TBM group were successfully genotyped for the *CYP2A6* copy number (call rate, 98%). The *CYP2A6* gene deletion occurred in 8 (17%) subjects and was in Hardy-Weinburg equilibrium (*P* = 0.394). There was no association of the *CYP2A6* copy number with the concentration of EFZ or metabolites in plasma or CSF either singly or in combination with the *CYP2B6* genotype. A single subject had the *CYP2A6* gene deletion in combination with a homozygous *CYP2B6* c.516G→T mutation; in this subject, the EFZ concentration was 6,319.5 ng/ml in plasma and 54.7 ng/ml in CSF.

### Addition of β-glucuronidase.

In the TBM group, the addition of β-glucuronidase did not significantly alter the concentrations of EFZ (not tested in the no-TBM group, as the levels were much lower). In contrast, the concentrations of 8OH-EFZ were much higher following β-glucuronidase addition. The ratio mean free/total concentration of 8OH-EFZ was 0.064 in plasma and 0.075 in CSF. Without β-glucuronidase, free 8OH-EFZ concentrations were low: mean concentrations were 87.3 ng/ml (95% confidence interval [CI], 63.8 to 122.5) in plasma and 3.7 ng/ml (95% CI, 2.7 to 5.7) in CSF.

Mean 7OH-EFZ concentrations in the TBM group with β-glucuronidase were 75.3 ng/ml in plasma and 3.5 ng/ml in CSF; without β-glucuronidase, 7OH-EFZ levels were below the lower limit of quantification. In the no-TBM group, mean 7OH-EFZ concentrations were 236.6 ng/ml in plasma and 1.3 ng/ml in CSF.

### Albumin ratio.

**FIG 3:**
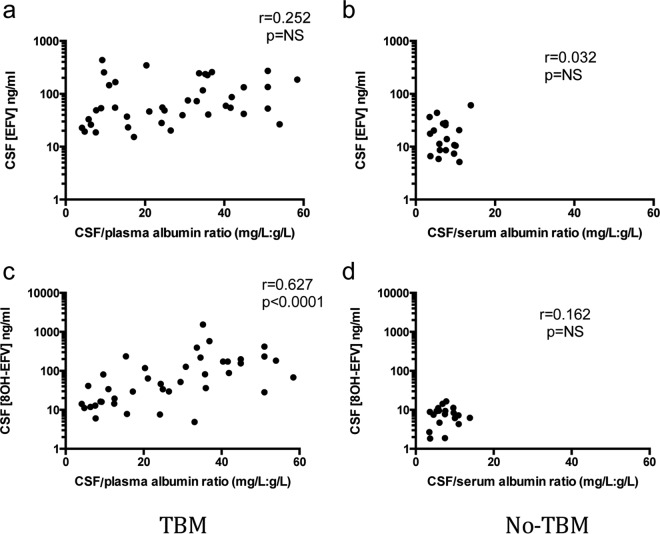
Relationship between degree of blood-brain barrier breakdown, as measured by the CSF/blood albumin ratio, and the CSF concentrations of EFZ and 8OH-EFZ.

The CSF/serum or plasma albumin ratio was abnormal in 35 (90%) subjects in the TBM group and 4 (21%) in the no-TBM group. In the TBM group, the CSF/plasma albumin ratio was positively correlated with the CSF 8OH-EFZ concentration (Fig. 3c). A nonsignificant trend with the CSF EFZ concentration was observed (Fig. 3a). In the no-TBM group, no correlation between the CSF/serum albumin ratio and the CSF EFZ or 8OH-EFZ concentrations was observed (Fig. 3b and d).

## DISCUSSION

We studied the concentration of EFZ and its metabolites in plasma and CSF and observed high CSF EFZ and 8OH-EFZ concentrations in patients with TBM, which were not observed in those without TBM. These differences could not have been explained by the higher doses of EFZ used in the TBM group (800 mg versus 600 mg in the no-TBM group) since plasma exposures were comparable across both study populations. We observed a strong correlation between plasma and CSF EFZ concentrations, and both were associated with the *CYP2B6* c.516G→T genotype. In contrast, the concentrations of the neurotoxic metabolite 8OH-EFZ were not related to plasma EFZ concentrations or the *CYP2B6* c.516G→T genotype but were correlated with the degree of blood-brain barrier breakdown measured by the CSF/plasma albumin ratio. These data confirm the findings of a recent publication from the ENCORE CNS substudy which demonstrated an association of the *CYP2B6* c.516G→T genotype with plasma and CSF EFZ concentrations but not with plasma and CSF metabolite 8OH-EFZ concentrations at doses of 400 mg and 600 mg (29). We demonstrate the same relationship at an EFZ dose of 800 mg, albeit when it is prescribed with rifampin, which induces the activity of *CYP2B6*.

The majority of EFZ metabolites in CSF were present as glucuronide conjugates. This is less likely to be due to CSF trapping of plasma glucuronide (the percentage of free compound was not significantly higher in CSF) and suggests that EFZ metabolites may be conjugated within the CNS. A number of UDP-glucuronosyltransferases have been demonstrated to be present in human brain tissue (30, 31). EFZ metabolites may have entered the CNS by crossing the blood-brain barrier or resulted from the CNS metabolism of EFZ. Functional *CYP2B6* and *CYP2A6* are present in the CNS, and expression has been shown to be inducible and subject to genetic variation (32–34). The significance of the fact that most 8OH-EFZ in CSF exists as a glucuronide conjugate is unclear; in particular, it is not known whether glucuronidated 8OH-EFZ induces the same neurotoxic effects as free compound or whether glucuronidation is in some way protective. We did not measure glucuronidation in the no-TBM group; however, a recent study in patients without TBM found similar high levels of 8OH-EFZ glucuronidation in CSF (35).

This is the first report of EFZ metabolites in the CSF of patients with TBM. CSF concentrations of EFZ and metabolites were higher in those with a loss of blood-brain barrier integrity due to TBM infection, and the concentrations were the highest in TBM patients with the greatest loss of blood-brain barrier integrity, as measured by the CSF/plasma albumin ratio. As EFZ is >99.75% protein bound in blood (36, 37), higher CSF EFZ concentrations may be due to leakage of the free fraction from plasma in those with a loss of integrity of the blood-brain barrier or due to increased trapping of EFZ in those with a higher albumin concentration in CSF.

CSF EFZ concentrations consistently exceeded *in vitro* neurotoxic concentrations in patients with a combination of TBM infection and the *CYP2B6* c.516G→T mutation (i.e., those with the GT or TT genotype, corresponding to intermediate or slow EFZ metabolizers, respectively). In contrast, CSF total 8OH-EFZ concentrations exceeded the *in vitro* neurotoxic concentration in the majority of subjects with and without TBM, regardless of genotype. This has implications for neuronal damage in patients with TBM, which could contribute to the overall neurological sequelae from this disease. Data from the recent ENCORE CNS substudy demonstrated an association of CSF 8OH-EFZ concentrations with symptoms at 1 year (29). The main limitation of our study is that we could not examine whether potentially neurotoxic CSF concentrations corresponded to clinical evidence of neurological dysfunction. There are several reasons why this was the case. In the TBM group, adverse neurological outcomes were attributed to TBM rather than drug neurotoxicity. Higher albumin ratios may reflect more severe TBM infection and, hence, confound any association of CSF 8OH-EFZ with clinical outcomes. The albumin ratio would be expected to decrease over time, which may coincide with clinical improvements. In the no-TBM group, detailed cognitive testing was not performed, and most patients in that group had a clinical indication for lumbar puncture which may have confounded associations with clinical outcomes. Further work is needed to determine the short- and long-term clinical consequences related to CSF 8OH-EFZ concentrations far exceeding *in vitro* neurotoxic levels, as this has important clinical implications. One question is whether EFZ should be avoided in those with impaired blood-brain barrier integrity, in particular, those with a neurological infection, such as TBM. However, as discussed above, such studies will be limited by difficulties in separating EFZ neurotoxicity from the effects of neurological infection. Another question is whether *CYP2B6* c.516G→T genotyping in clinical practice would lower the incidence of neurocognitive side effects. Our data suggest that avoiding EFZ in those with the GT or TT genotype would not alter CSF 8OH-EFZ concentrations and, hence, may not be an effective strategy.
